# May I Smell Your Attention: Exploration of Smell and Sound for Visuospatial Attention in Virtual Reality

**DOI:** 10.3389/fpsyg.2021.671470

**Published:** 2021-07-22

**Authors:** Nicolò Dozio, Emanuela Maggioni, Dario Pittera, Alberto Gallace, Marianna Obrist

**Affiliations:** ^1^Politecnico di Milano, Department of Mechanical Engineering, Milan, Italy; ^2^Sussex Computer-Human Interaction Lab, Department of Informatics, University of Sussex, Brighton, United Kingdom; ^3^Department of Computer Science, University College London, London, United Kingdom; ^4^Ultraleap Ltd., Bristol, United Kingdom; ^5^Mind and Behavior Technological Center - MibTec, University of Milano-Bicocca, Milan, Italy

**Keywords:** virtual reality, smell, sound, multisensory, visuospatial attention

## Abstract

When interacting with technology, attention is mainly driven by audiovisual and increasingly haptic stimulation. Olfactory stimuli are widely neglected, although the sense of smell influences many of our daily life choices, affects our behavior, and can catch and direct our attention. In this study, we investigated the effect of smell and sound on visuospatial attention in a virtual environment. We implemented the Bells Test, an established neuropsychological test to assess attentional and visuospatial disorders, in virtual reality (VR). We conducted an experiment with 24 participants comparing the performance of users under three experimental conditions (smell, sound, and smell and sound). The results show that multisensory stimuli play a key role in driving the attention of the participants and highlight asymmetries in directing spatial attention. We discuss the relevance of the results within and beyond human-computer interaction (HCI), particularly with regard to the opportunity of using VR for rehabilitation and assessment procedures for patients with spatial attention deficits.

## Introduction

In our everyday life, we tend to underestimate the importance of smell as a source of information and interaction with our environment. Smell can evoke memories more intensively than any other modality (Herz and Schooler, 2002; Obrist et al., 2014), and it can be used to convey meaning (e.g., warn us of danger) (Obrist et al., 2014), or promote instinctive behaviors (e.g., avoidance) (Stevenson, 2009). Humans selectively shift their attention based on the presence of certain smells in their surrounding space by modulating their distance to the source and based on the perceived pleasantness of an odor (Rinaldi et al., 2018). While the effect of smell on spatial attention is increasingly studied in psychology and neuroscience, its study within HCI is still in its infancy.

The spatial design features of smell are increasingly recognized for virtual reality (VR) applications. For example, VaiR (Rietzler et al., 2017) and Head Mounted Wind (Cardin et al., 2007) are both extending the VR headset with wind and thermal stimuli, while Season Traveler (Ranasinghe et al., 2018) also integrates olfactory stimuli to increase the sense of the presence of users in a multisensory VR experience. While there is a growing interest in exploring the potentials around smell in VR to enrich user experiences with olfactory stimuli (Carulli et al., 2018; Risso et al., 2018; Bordegoni et al., 2019) and in designing wearable olfactory interfaces [e.g., Wang et al. (2020), Amores and Maes (2017), and Amores et al. (2018)], the study of smell in relation to its spatial features is still limited, [e.g., Maggioni et al. (2020), Shaw et al. (2019), and Kim and Ando (2010)]. Hence, here we specifically investigated the effect of smell presented under unimodal and multimodal conditions for catching the attention of users during a visual exploration task in VR.

The participants were asked to search for specific visual targets among distractors in a virtual environment. We adopted three experimental conditions (smell, sound, and smell and sound) and a control condition (vision only). To enable this investigation, we created a VR implementation of the Bells Test (Gauthier et al., 1989), an established neuropsychological test used to assess attentional and visuospatial disorders (Ferber and Karnath, 2001; Azouvi et al., 2002). The Bells Test is widely used in clinical settings for the diagnosis of unilateral spatial neglect (USN), which describes the inability of certain patients with brain damage to respond to stimuli presented on the contralesional side of their space.

Based on the results from the experiment with the 24 participants, we show that performance was higher when multimodal stimulation was used. That is, using a combination of olfactory and auditory stimuli was significantly more effective in capturing the visuospatial attention of the participants toward the right side of the VR exploration space. Interestingly we found asymmetries in the spatial orientation effects. That is, stimulation coming from the left side of space resulted in a higher performance toward the left, regardless of the three experimental conditions. We discuss these findings in light of the growing efforts toward the development of multisensory HCI applications, especially in the context of promoting VR as an effective tool both for the assessment and rehabilitation of brain damage disorders related to spatial attention.

## Related Study

In this section, we discuss the relevant related studies on attention and spatial attention, specifically through the lens of multisensory stimulation and olfactory research, which is increasingly recognized within HCI. We highlight the potential of VR not only for the design of multisensory VR experiences through the integration of smell but also as a tool for implementing and adapting traditional assessment methods of spatial neglect.

### Attention and Spatial Attention Using Visual, Auditory, and Tactile Stimuli

When considering attention, one of the simplest distinctions that can be made is between top-down and bottom-up processes. The first is referred to the voluntary allocation of attention on specific information or features, while the latter is referred to the automatic attention shift triggered by salient sensory stimulation (Corbetta and Shulman, 2002; Pinto et al., 2013). Spatial attention focuses on prioritizing spatial locations in the environment, and it includes both the top-down and bottom-up processes. The brain area mainly involved in the visuospatial processing and movement orienting is the superior colliculi (Krauzlis et al., 2013). This area is also strongly involved in the integration and processing of all the multisensory inputs perceived from the external environment (Meredith and Stein, 1986). Attentional shifts in one modality are usually accompanied by the integration of other modalities (Driver and Spence, 1998) (e.g., a sudden movement followed by a sound perceived from the same spatial area causes the integration of vision and audition). Neural correlates for this process include the ventral frontoparietal network strongly lateralized to the right cerebral hemisphere (Corbetta and Shulman, 2002).

The attentive system would seem to be also responsible for the recognition of multisensory objects by binding simultaneously multimodal signals across space (Busse et al., 2005). Specifically, the integration of different sensory modalities has been proven to have a strong effect when related to spatial attention. For example, Santangelo (Santangelo and Spence, 2007; Santangelo et al., 2008) explored the use of multisensory cues to capture spatial attention during low and high perceptual load tasks, and their results appear to indicate that only bimodal cues effectively captured spatial attention regardless of any increase in perceptual load. Moreover, Keller (2011) made a comparison between visual and olfactory attention features and highlighted similarities between the two modalities, especially when allocating attention in a specific time section and on specific features (e.g., odor pleasantness).

The importance of understanding the mechanisms that influence the attention of users, especially related to its visuospatial characteristics, is increasingly recognized when designing interactive systems (e.g., in computer vision, see review by Nguyen et al., 2018). More specific to VR, Dohan and Mu (2019) implemented a gaze-controlled VR game that deployed an eye tracking system to better explore the attention and behavioral patterns of users, while Almutawa and Ueoka (2019) explored the influence of spatial awareness in VR, especially during the transition from the real world to VR environments.

### The Role of Smell Stimulation for Spatial Attention

Despite the predominance of studies on other sensory modalities, there is a growing body of research on smell and cross-modal correspondences studies, especially on smell and sound (Belkin et al., 1997; Crisinel and Spence, 2012; Deroy et al., 2013). Von Békésy (1964), for example, suggested the existence of similarities in the way the brain processes the direction of auditory stimuli and odors. Results of this work showed that the olfactory system is also capable of determining the direction of a scent in a way that is similar to the auditory system, but with lower spatial sensitivity, by recognizing time differences between the two nostrils in the order of 0.3 msec. Considering similar principles, Porter et al. (2007) demonstrated that humans can navigate space by scent-tracking. Moreover, there is evidence supporting the existence of a link between olfaction and vision, highlighting the role that smell plays in attracting visual attention. An example is provided by the work of Seigneuric et al. (2010), who found that in a visual exploration task, smell influences visual attention and, more specifically, facilitates the recognition of an object that is associated to the odor perceived. All these findings encouraged greater consideration of the possible role that smell can play on spatial attention when combined with different sensory modalities.

Few studies have recently investigated the relationship between attention and multisensory stimulation, such as between vision, touch, and audition (Spence et al., 1998; Van Hulle et al., 2013); attention and tactile information (Spence, 2002; Spence and Gallace, 2007); and audition and vision (McDonald et al., 2000; Gallace and Spence, 2006). These studies provide evidence that multisensory stimuli lead to faster and stronger responses than single sensory stimuli. The enhanced multisensory response is not simply due to the additive effects of concurrent sensory information. Multisensory stimulation often elicits more accurate responses than the expected response predicted by additive models of unimodal stimuli (Colonius and Diederich, 2003; Murray et al., 2005; Spence and Ho, 2008; Lunn et al., 2019). The concept of “super-additivity,” because of the perception and the integration of stimuli from different sensory modalities, allows to derive three basic rules (Holmes and Spence, 2005). Researchers found that to obtain an increase in neural activity, and consequently a stronger and faster response, multisensory stimulation must come from similar space locations, must be perceived almost at the same moment, and at least one of the stimuli must be only weakly effective in evoking a neural response (Holmes and Spence, 2005).

### Technology Advances to Enable the Study of Spatial Attention With Smell

Recent developments have resulted in systems such as VaiR (Rietzler et al., 2017), Head Mounted Wind (Cardin et al., 2007), and Season Traveler (Ranasinghe et al., 2018), which illustrate the advances in multisensory technology. Studies of systems, such as VaiR (Rietzler et al., 2017) and Head Mounted Wind (Cardin et al., 2007), which are wearable displays using arrays of fans or pneumatic air nozzles attached to users as extensions of a VR headset, have shown that participants experienced an increased sense of presence and can correctly detect air-flow directions but do not account for the sense of smell. Season Traveler (Ranasinghe et al., 2018) extends wind and thermal stimuli through olfactory stimuli and confirmed the previous results regarding an enhanced sense of presence in a virtual environment. In this case, the scented air was released near the nose of the user to increase the sense of presence in a multisensory VR experience but was not studied with regard to its spatial features. Indeed, while there is a growing interest in designing wearable olfactory interfaces (e.g., Amores and Maes, 2017; Amores et al., 2018; Wang et al., 2020), and enriching user experiences with olfactory stimuli (Bordegoni et al., 2019; Micaroni et al., 2019), the study of smell in relation to its spatial features is still limited (e.g., Kim and Ando, 2010; Shaw et al., 2019; Maggioni et al., 2020).

Most recently, Maggioni et al. (2020) not only highlighted the spatial design features for the sense of smell but also illustrated its effect in three application scenarios, including two VR implementations. Most relevant for this study is a described VR implementation that focuses on the ability of users to locate the source of an olfactory stimulus in a 360° virtual space. The authors compared smell with sound stimuli and combined these two sources of information. Their results showed an interaction effect between the stimulation modality and position of users on the accuracy in locating the source [F_(2, 12)_ = 18.6, *p* < 0.01, η^2^ = 0.75], and provide valuable information about the performance of users in localizing olfactory and auditory spatial cues. More specifically, the accuracy of users in the front position in VR was comparable across all modalities but was significantly different between olfactory and auditory stimuli, and olfactory and audio-olfactory in the back position (i.e., front position refers to the space when the cue is presented within 180° centered on the head direction of the participant, and back position when outside 180°). Olfactory stimuli together with auditory stimuli can play an even more important role in the navigation of VR environments where all the interactions are mainly based on audiovisual stimulation, which can easily lead to overwhelm the attention of users. This study provides valuable information on the importance of carefully choosing the scent-delivery parameters when exploring the spatial features of smell, such as we do in our smell-enhanced VR implementation of The Bells Test, a well-established neuropsychological test.

### VR Implementation of the Bells Test

**Figure 1:**
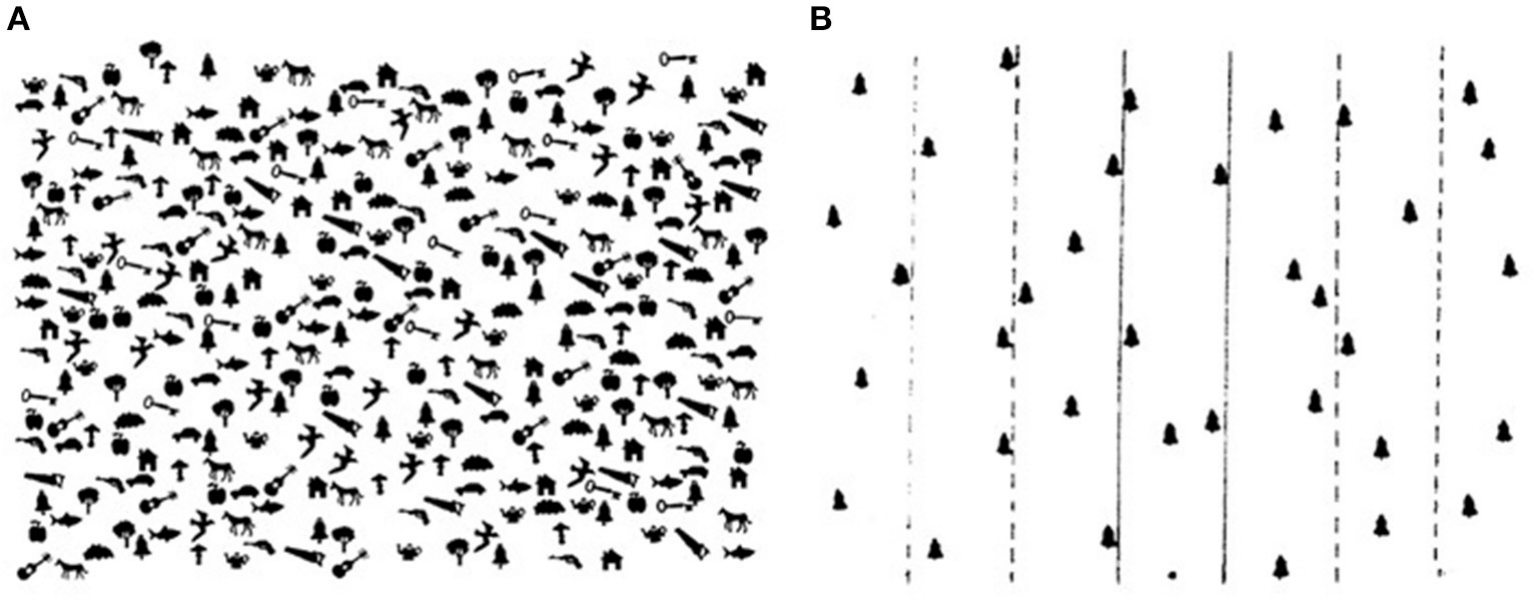
**(A)** Bells Test and **(B)** its target distribution (Gauthier et al., 1989).

Visual exploration tasks, such as line bisection task (i.e., a task where participants have to highlight the middle point of a horizontal line presented on a sheet located in front of them) or cancellation task (i.e., a task where participants have to search and highlight visual targets among distractor figures), are commonly used in neuropsychology for the assessment of visuospatial deficits (Plummer et al., 2003). The Bells Test (Gauthier et al., 1989) (Figure 1) is widely used for the assessment of unilateral spatial neglect (USN).

Neglect is generally defined as “the failure to report, respond, or orient to stimuli presented to the side opposite a brain lesion, when this failure cannot be attributed to either sensory or motor defects” (Heilman et al., 1984). Neglect is mainly due to lesions involving the right inferior parietal and adjacent temporal lobe (Vallar and Perani, 1986) and occurs in ~30% of patients who had a stroke (Nijboer et al., 2013). There is also evidence of ipsilateral neglect, where the neglected side of the space corresponds with the lesioned brain hemisphere (Weintraub and Mesulam, 1987). In addition, patients with neglect usually show a lack of awareness for their disorder (anosognosia) (Gialanella et al., 2005). For example, when asked to copy an image located in front of them, they copy only the right half of it and, in the same way, they may eat only the right part of their food (reporting that their performance is correct).

Cancellation tasks like the Bells Test are commonly performed during the assessment of this deficit, and patients usually show behavioral patterns of hypo-attention on the contralesional side (i.e., left) and hyper-attention on the ipsilesional side (i.e., right) (Albert, 1973; Rapcsak et al., 1989; Plummer et al., 2003). Participants are instructed to search and highlight as many bells as they can see among visual distractors (see Figure 1). This is traditionally done with paper and pencil, with the additional instruction not to move the sheet located in front of them. In this way, neuropsychologists can understand the exploration path followed by the patient, to assess possible visuospatial and attentional deficits such as USN.

Here, we present a VR implementation of the Bells Test, augmenting the visual search with olfactory and auditory stimulations, delivered individually or combined, to explore the effect of these modalities on the visuospatial attention and performance of the participants in VR. We maintained the same features of the Bells Test, as it would be used in clinical settings with neuropsychological patients; however, we adapted the test to cover a larger canvas and provide an immersive experience for participants without attentional or visuospatial deficits. To do so, we increased the number of targets (i.e., from 35 to 75) and distractors (i.e., from 280 to 520) of the Bells Test to cover a 180° VR exploration space that simulated a distance between the participant and the semicircle space of ~2 m. As mentioned above in Section Technology Advances to Enable the Study of Spatial Attention With Smell, we followed the recently published study by Maggioni et al. (2020) on the accuracy of the participants in the front and back position in VR when using olfactory stimuli, making us focus only on the front position, within 180° centered on the head direction of the participants.

**Figure 2:**
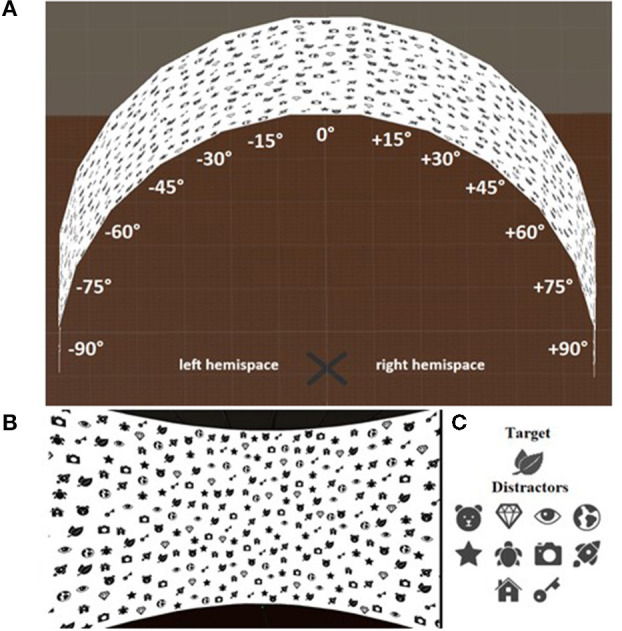
Illustration of the VR implementation of the Bells Test with **(A)** the 13 sections and **(B)** from the point of view of the participants. **(C)** Target and distractor items used in the VR implementation.

We divided the VR space into 13 sections equivalent in height and width, consisting of a central section and six sections for each of the left and right hemispaces (see Figure 2). The central section was set at 0° rotation, perpendicular to the participant point of view during the visual exploration task, while for the left and right hemispaces, each of the six sections increased its rotation on the vertical axis by 15° until they reached +90° for the right hemispace and −90° for the left hemispace. Each section contained five targets and 40 distractors. The VR environment was rendered in order to prevent the participants to perceive the edges of the 13 sections that constituted the 180° exploration space and gave the illusion of a regular semicircle (Figure 2). This solution avoids the adoption of a section-by-section exploration procedure and promoted, instead, the exploration of the entire space without visual bias. The VR scenario was created using Unity 3D Software (version 2018.2.5), and the participants were asked to perform the visual exploration task wearing an HTC Vive VR headset.

For the user study, we selected peppermint as the olfactory stimulus. Peppermint scent has been proven to have an arousing effect on the central nervous system and has been used in a previous study on attention (Warm et al., 1991; Dember et al., 2001), as well as on workload, work efficiency, and alertness, for example in driving tasks (Raudenbush et al., 2009). To ensure semantic congruency between visual and olfactory stimuli, based on a prior study (Raudenbush et al., 2009), we replaced the traditional “bells” in the Bells Test with a new target symbol (i.e., two small mint leaves, see Figure 2).

## Materials and Methods

The study aimed to explore the effect of olfactory and auditory stimulation on visuospatial attention. We used the above-described implementation of the Bells Test while integrating the different modalities and compare the performance of the participants under three experimental conditions: smell, sound, and smell and sound stimuli. We used a visual-only control condition, without any additional sensorial stimulation. Thus, we had a total of four conditions as part of the study design. The study was approved by the University Science and Technology Cross Schools Research Ethics Committee. Each participant gave written informed consent after being instructed about the study procedures and completing an olfactory assessment test (Nordin et al., 2003) to exclude people with smell dysfunctions, adverse reactions to strong smells, respiratory problems, flu, serious head trauma, or brain disorders.

### Study Design and Procedure

This was a mixed model study design with three experimental conditions and a visual-only control condition (without any additional sensorial stimulation) as a within-participants factor, and the stimulation origin side as a between-participants factor. The participants were asked to complete the visuospatial exploration task in VR as fast as possible (i.e., “find the target item ‘mint leave”'). Each condition followed the same rules as the control condition but with the introduction of smell (*Condition I*), sound (*Condition II*), and combined smell and sound (*Condition III*) stimulation during the exploration task. Therefore, for each participant, we tested the unisensory effects in condition I and II (i.e., olfactory and auditory only) and then the multisensory effects in condition III (i.e., combined smell and sound). The stimulation origin side for each condition was counterbalanced between the participants. The exploration space was limited to a 180° field of view to avoid participants turning around and invert the origin of the stimuli, and in line with a prior study (Maggioni et al., 2020) that showed the effectiveness of focusing on the front position (see explanation in Section Technology Advances to Enable the Study of Spatial Attention With Smell). The participants were asked to perform the visual exploration task wearing an HTC Vive VR headset. At the beginning of each exploration task, the participants were instructed to wear the headset and to reach a mark located in the middle of the virtual semicircle space (see Figure 2A). While performing the task, they were allowed to freely move their head and rotate their body, but they were asked to remain on the mark.

**Figure 3:**
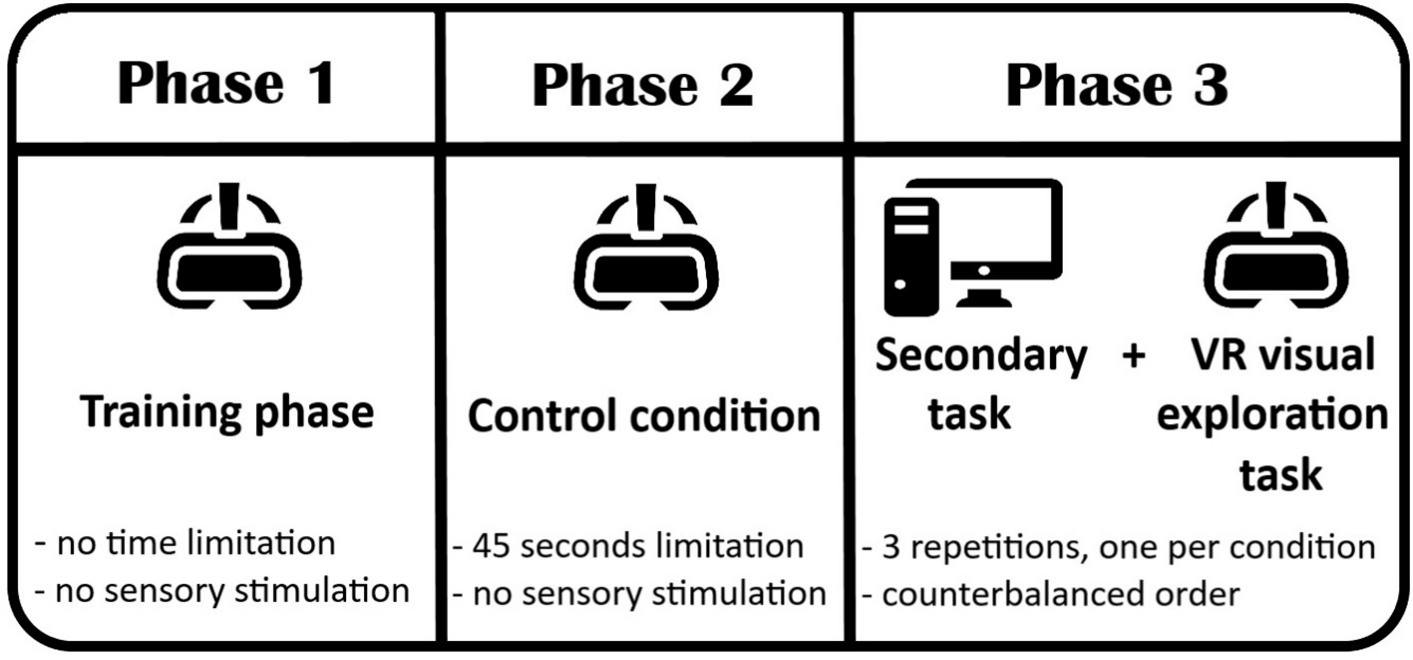
Overview of the three main study phases. After the training phase and the control condition of the VR visual exploration task, the participants proceeded with the three experimental conditions starting from the Secondary task followed by the VR exploration task. The conditions were counterbalanced between the participants.

For the investigation of the effect of unimodal and multimodal stimuli on visuospatial attention in VR, we used peppermint (i.e., essential oil from Holland and Barrett) as the olfactory stimulus due to its above-described effect on the central nervous system and its prior use in studying attention (Warm et al., 1991; Dember et al., 2001). As an auditory stimulus, we opted for a simple sequence of three piano G tones, where the first and third tones were the same, while the second was an octave higher of the other two. This sequence was used to avoid any correlation due to their pitch following prior studies (see Ben-Artzi and Marks, 1995; Carnevale and Harris, 2016; McCormick et al., 2018). The study was divided into three phases (see an overview in Figure 3).

The participants started with the training phase (Phase 1) to familiarize themselves with the VR exploration task without any time constraints or any sensory stimulation and distractors, only with visual targets. Phase 1 was then followed by the control condition (Phase 2) and finally by the main part of the experiment (Phase 3), composed of two parts: a secondary task and the visuospatial exploration task with all three conditions. The order of the conditions and the origin of the stimulation (left or right hemispace), excluding the initial control condition, were counterbalanced using a Latin square design, as it was the position of targets and distractors in each of the 13 sections among task conditions. Each of those three phases is described in detail below.

#### Phase 1

During the training phase, the participants could familiarize themselves with the exploration task, the controller functionality, and the VR environment. They were instructed to find and select specific targets (i.e., small figures of mint leaves). They selected the target by pointing at it with a VR reproduction of a green ray that originated from the bottom of the HTC controller, and by pulling and releasing the trigger to highlight it. Visual instructions on the use of the controller were provided to the participants. They were also informed of the time constraint in the actual testing phase that would follow later. In this phase, there were no distractors, the targets were highlighted in red, and no time limit was set. At the end of the training phase, the participants could restart the training until they were comfortable with the task and interaction. This phase was important to counteract any possible bias due to novelty effects introduced by the visual exploration task and the VR environment. When a participant was ready to proceed, we started with the control condition.

#### Phase 2

Here, the participants proceeded with the control condition (i.e., the visual-only exploration task). They were instructed to find and select the targets (i.e., mint leaves) among the distractors (as shown in Figure 2). They selected the target by pointing at it with the VR reproduction of a green ray that originated from the bottom of the HTC controller, and pulling and releasing the trigger to highlight it. In section Measures and Hypotesis we describe the measures employed to evaluate the performance of the participants.

#### Phase 3

This final phase comprises a secondary task followed by the VR visual exploration task with all three experimental conditions. The secondary task condition followed the same counterbalanced order of the VR task and was carried out before each of the three experimental conditions to allow the participants to familiarize themselves with the sensory stimulations they would experience later in the VR task. Therefore, the secondary task condition always corresponds to the following VR experimental condition. This step aimed to reduce the novelty effect and biases due to the introduction of the olfactory and auditory stimuli that characterize the experimental conditions of the VR visual exploration task, and its data were not further analyzed. The secondary task was performed on a desktop computer monitor, where a sequence of figures, as used in the VR Bells Test implementation (see Figure 2), were presented on a monitor (22” diagonal). The monitor was located on a desk at a distance of 150 cm from the participants. Behind the monitor, we located a speaker to deliver the sound stimuli; and, on the top of the monitor, there was a nozzle connected to the smell delivery device. For both trials, the participants had to press on a keyboard the “Y” key if in the set of images the target was present, and the “N” key if the target was not included in the set of images. The target figure was anticipated by smell (condition I), sound (condition II), or smell and sound (condition III) stimuli.

**Figure 4:**
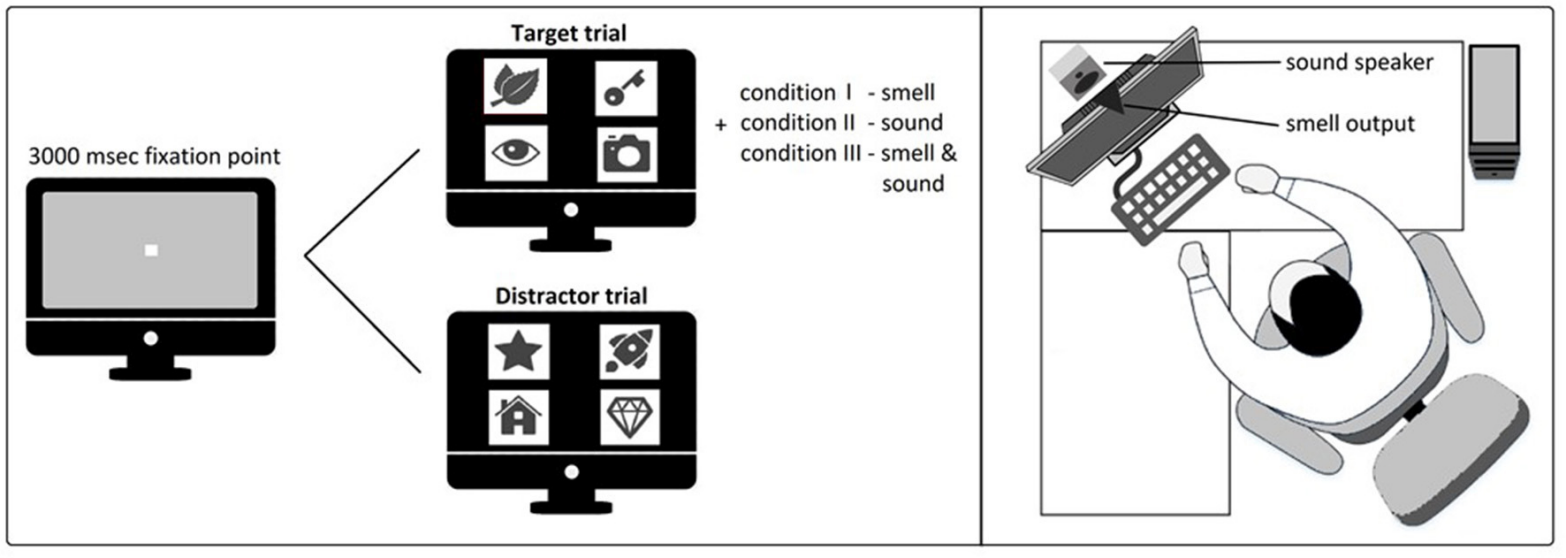
Overview of the secondary task presentation and setup.

This secondary task was composed of a randomized presentation of 30 distractors trials and 10 target trials. Each trial differs from the other based on the combination of images and their position. Each trial starts with a 3,000 ms. fixation point (white square sized 10 × 10 pixels), followed by a presentation of a set of four images, located as shown in Figure 4. In the distractor trials, we presented four random distractors, and in the target trials we presented three random distractors and the target (the same mint leaves icon used in the VR exploration task). For the target trials, the fixation point was accompanied by the same sensory stimulation used for the VR exploration task, according to the three experimental conditions: single smell presentation with 1 s delivery time (condition I), single presentation of the sound sequence (condition II), and combination of smell and sound (condition III).

After completing the secondary tasks, the participants were verbally introduced to continue with the visual exploration task in VR (all three conditions in counterbalanced order between participants). Each visual exploration session was limited to 45 s. During a pilot study with five participants, we observed that this time interval was long enough to allow the participants to explore almost all the VR exploration space, but it was difficult for them to select all the targets in both hemispaces. This choice allowed us to observe in greater detail the differences between the two hemispaces because of the experimental conditions.

### Sensory Setup and Stimuli Presentation in VR

**Figure 5:**
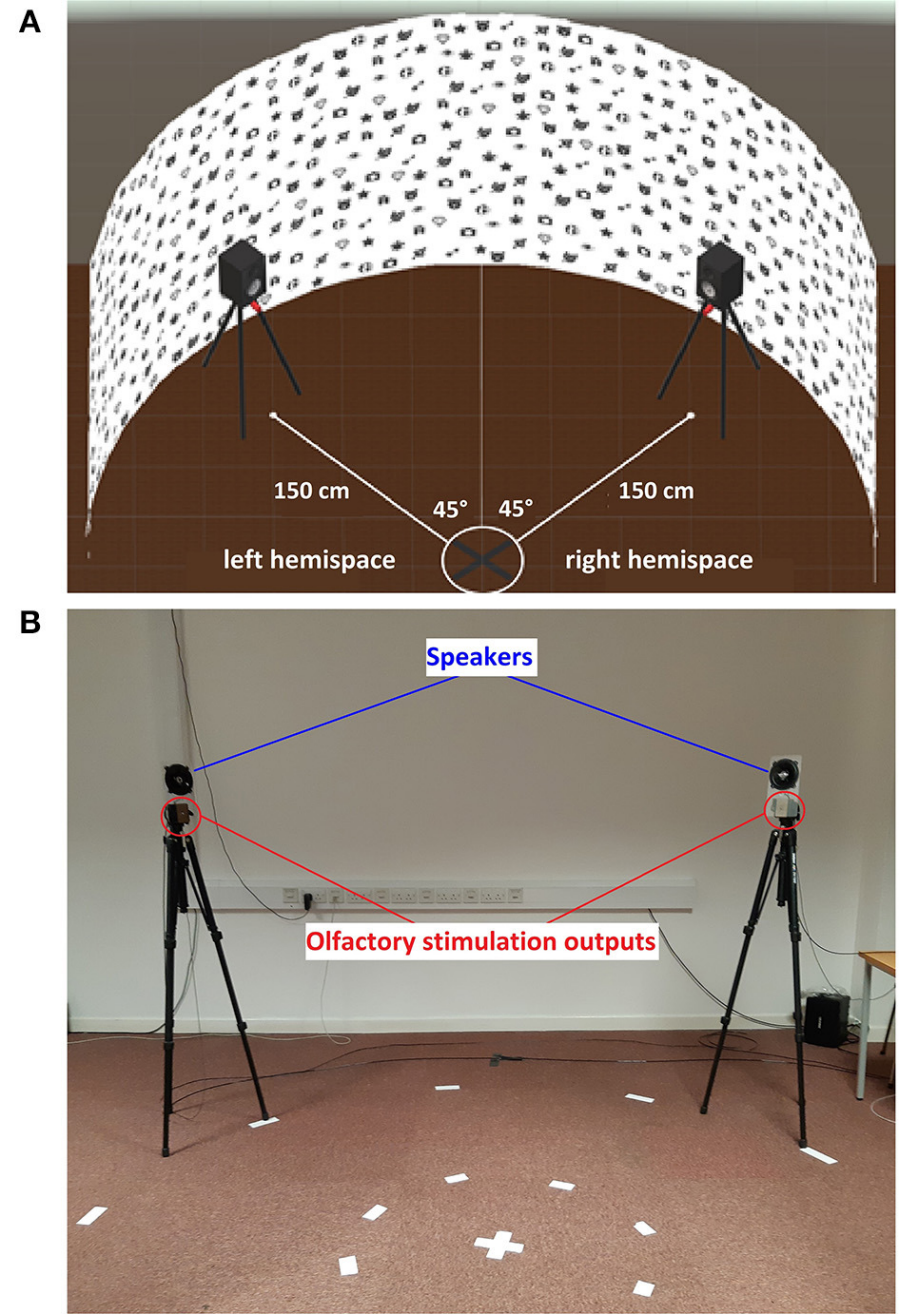
Schematic representation of the VR environment. **(A)** The curved white virtual wall used to implement the Bells test inside the VR headset. **(B)** The real world location of the olfactory (red) and auditory (blue) stimulation outputs integrated on top of a tripod.

The stimuli origin side was counterbalanced between the left or right hemispace, between the participants. The delivery point for olfactory and auditory stimuli was set at ±45° from the head of the participants at a distance of 150 cm (Figure 5). We set the origin of the stimuli at ±45° to drag the attention of the participants in the middle of the right or left hemispace. This also allows the participants to continue the visuospatial exploration without being distracted toward the extreme edge of the hemispace.

The olfactory stimuli were administered using a custom-made computer-controlled scent delivery device, previously used in other research studies (Maggioni et al., 2018, 2020; Cornelio et al., 2020). The delivery device contains three electro-valves (4-mm Solenoid/Spring pneumatic valve) that regulates the airflow (on-off) from an ultra-low noise oil-free compressor with a storing tank (8 Bar maximum capacity, 24 L, 93–78 L/min at 1–2 Bar, Bambi Air, Birmingham, United Kingdom). The compressor supplies a regulated air flow (max 70 L/s) through 4-mm plastic pipes passing by the electro-valve and arriving in glass bottles that contained the essential oil. The airflow was set at a constant pressure of 1.5 Bar-L/min, through an air regulator. The smell reached the participants through a 3D-printed single-channel nozzle. Each valve was connected with a jar of essential oil, so we have one for the left hemispace, one for the right hemispace, and one for the secondary task. The nozzles for the smell output were anchored in laser cut plates and attached to the sound speakers that were mounted on and attached to two tripods. Thus, the olfactory and auditory stimuli shared the same output position.

With the setup shown in Figure 5, the olfactory stimulus was reaching the participants in an average of 6 s, based on a pilot study we have conducted with five participants. During the pilot study, the participants were instructed to report when they started to perceive the olfactory stimuli by pressing the trigger of the HMD controller. With the same procedure, we have tested additionally the auditory stimulus duration for synchronizing auditory and olfactory stimuli for condition III in the study. The delivery time for the olfactory stimulus was 1 s with delivery intervals of 5 s and automatically triggered at the beginning of the visual exploration task. The delivery was not continuous to avoid any possible habituation effect and environmental saturation. For the auditory stimulus, the duration of the sequence of three piano G tones was also set at 1 s and repeated every 5 s until the end of the task. Hence, the auditory and olfactory stimulations were synchronized, with the auditory stimulation starting after 5 s with a duration of 1 s, matching the 6 s needed for the smell to reach the user.

### Measures and Hypothesis

To assess the performance of the participants, we collected the following data at the end of each condition. All the data were automatically recorded by the script implemented in Unity. We measured (a) the number of targets selected (i.e., mint leaves) with details about their location (left or right hemispace, section of appearance, see Section Materials and Methods); and (b) the starting point (which of the 13 sections in the left or right hemispace) and the exploration path followed by the participants during the VR visual exploration task, combined with data collected through the VR headset and related to the head position of the participants.

Based on a prior study, we had the following two hypotheses:

H1: the introduction of olfactory and auditory stimulation will draw the attention of the participants to the hemispace from which they are delivered.
H2: the combination of both stimuli (i.e., additive effect of multisensory stimulation explained in section The Role of Smell Stimulation for Spatial Attention) will have a stronger effect than the single sensory stimulation on the performance of the participants.

## Results

An *a priori* statistical power analysis to estimate sample size using G^*^Power was performed. We set the repeated measures ANOVA with the four conditions as a within-participants factor (i.e., control, I, II, and III) and between-participants factor on the stimulation side. Approximately 21 participants are required to obtain a power of 0.95, an alpha level of 0.05, and a medium effect size (f = 0.25) (Faul et al., 2007; Lakens, 2013).

The study involved a total of 24 participants (*M* age = 28.96, ± 5.14 years; five females; five left-handed). All the participants had a normal or corrected-to-normal vision. Based on the olfactory assessment test result, nobody reported any olfactory dysfunctions preventing them from participating in the experiment.

To test the hypotheses (see Section Measures and Hypothesis), we considered data related to the number of selected targets and their position in the exploration space (i.e., left or right hemispace). The analysis was performed on IBM SPSS 26. First, we explored the data through a normality test [Shapiro–Wilk test (Sheskin, 2011)]. The distribution of the targets selected for both sides in the three experimental conditions followed a normal distribution (*p* >.05). Therefore, we applied parametric analyses. We performed a general linear model (GLM) multivariate analysis with the number of targets selected in the left and right hemispaces (visuospatial attention performance) as dependent variables with experimental conditions (i.e., control, smell, sound, and smell-sound) and the origin side of sensory stimulation (i.e., the left or right side) as independent fixed factors.

**Figure 6:**
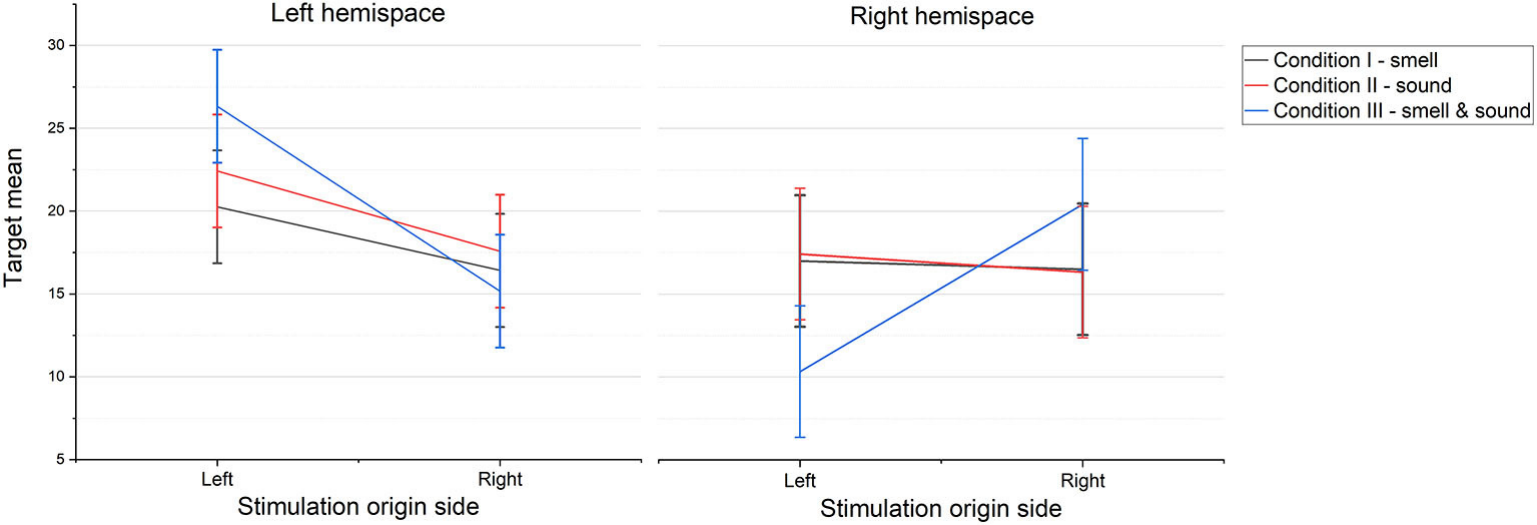
Mean score of the participants during visuospatial exploration task on the left and right hemispaces under the three experimental conditions for the two origin sides (left or right) of the sensory stimulation. Error bars represent the standard errors of the means.

The results showed a significant main effect of the origin side of sensory stimulations (i.e., left or right) on the visuospatial attention performance of the participants [*F*_(1, 89)_ = 11.23; *p* = 0.001] particularly on the left hemispace (*M* left = 23 SD = 7.58, *M* right = 16.39; SD = 9.33) (H1). This means that the participants explored more accurately the left side of the VR space when the sensory stimulation originated from the left side, independently from the type of stimulation (experimental condition). Furthermore, as shown in Figure 6, the results showed a significant interaction effect of experimental condition with the origin side of sensory stimulations on the visuospatial attention performance of the participants on the right hemispace [*F*_(2, 89)_ = 1.36; *p* = 0.037]. Nevertheless, an observation of the graphs (Figure 6) would seem to suggest a stronger difference in condition III, where olfactory and auditory stimuli were combined. Multiple comparisons (Bonferroni corrected) showed that for the right hemispace, there was a significant difference (*p* = 0.004) in condition III between the targets selected when the stimulation came from the left (*M* = 10.33; *SD* = 7.87), and those selected when the stimulation came from the right (*M* = 20.42; *SD* = 7.98). This result seems to support the hypothesis of a stronger effect of multisensory stimulation compared with unimodal stimuli.

To verify which experimental condition drove the visuospatial attention of the participants, we calculated the hemispace dominance (i.e., the hemispace with the highest number of targets selected) for the control condition (no stimulation) and for each experimental condition. We then considered the congruency between the origin side of the sensory stimulation and the hemispace dominance of the participants in each condition (i.e., whether participants found more target stimuli in the hemispace that matched the origin of the sound and smell). Performing a chi-square analysis on the congruency frequencies, the results showed no significant difference between hemispace dominance (left or right) in unisensorial condition [i.e., smell **χ**2_(1)_ = 0.67, *p* > 0.05, or sound **χ**2_(1)_ = 0.01, *p* > 0.05] and in the control condition [i.e., no stimulation χ*2_(1)_ = 0.17,* *p* > 0.05]. Instead, we found a significant difference for the multisensory stimulation (i.e., smell and sound together [**χ**2_(1)_ = 8.17, *p* = 0.004]). The hemispace dominance tended to be congruent with the origin of the stimulation during the multisensorial condition (H2) (see Table 1).

**Table 1:** Chi-Square analysis on participants' hemispace dominance and congruency^a^.

**Dominance congruency with the origin side of the sensory stimulation**	**Condition I smell**	**Condition II sound**	**Condition III smell & sound**
Congruent	14	12	19
Incongruent	10	12	5
Chi-Square	0.67	0.001	8.17**

* Hemispace dominance is related to the hemispace (left or right) with the highest number of targets selected in each condition. Congruency is defined as the number of times hemispace dominance matched the hemispace origin of the audio and smell stimulation*.

** *p < 0.01*.

## Discussion

In this section we discuss the principal effects of multisensory stimulation on visuospatial attention, focusing specifically on the spatial differences highlighted by the results. We highlight the potential of multisensory stimulation and VR for enhancing the design of future HCI tools through the integration of smell. Finally, we consider the opportunity of using VR as a tool for implementing and augmenting classical neuropsychological rehabilitation with multisensory stimulations.

### Multimodal Stimulation Effect on Visuospatial Attention

The study presented here explored the effect of auditory and olfactory stimulation on visuospatial attention and found evidence of a strong effect obtained by the integration of these two modalities related to the right hemispace of the exploration space. In the left hemispace, we observed a significant main effect of the origin of sensory stimulations (±45° left or right), independently from the sensory modality deployed.

Considering the prevailing occidental population that participated in the study, this difference between the two hemispaces could be explained by lexical-cultural aspects, since all of the participants were trained since childhood in a left-to-right orientation in reading and writing. There is evidence in the literature that supports this hypothesis. At the end of the 1960s, a series of studies had demonstrated that a point can be more accurately located when presented to the left visual field (Kimura, 1969). Another study (Vaid and Singh, 1989) explored asymmetries on the perception of visual stimuli with four different groups of participants composed of left-to-right readers, right-to-left readers, left-to-right and right-to-left readers, and illiterates. Their results revealed a significant left hemifield preference only for the left-to-right readers, while they did not find any reliable differences between left- and right-handers.

These results could be further explained by the pseudoneglect phenomenon (Bowers and Heilman, 1980). Similar to neglect, pseudoneglect is predominantly observed during line bisection tasks in subjects without visuospatial or attentional deficits and is characterized by a systematical tendency to mismatch the midpoint of a line. Specifically, patients with neglect usually make errors toward the right side of the line, while non-neglect subjects tend to locate the midline toward the left (Bowers and Heilman, 1980; McCourt and Jewell, 1999; Jewell and McCourt, 2000). All these findings support natural facilitation towards the left exploration space, leading to an increased attentional focus on the left hemispace. This could explain why the participants in this study appeared to be less influenced by the stimulation used to catch their attention toward the left hemispace, while for the right hemispace only multisensory stimulation seemed to have the power to influence the performance of the users due to the additive stimulation value of both sound and smell combined (Colonius and Diederich, 2003; Murray et al., 2005; Spence and Ho, 2008; Lunn et al., 2019). Interestingly, in Voinescu et al. (2020), the authors concluded that auditory stimulation is recommended for VR tasks requiring frequent interaction and responses to relevant information, showing a better performance when compared with visual stimuli. In this work, in contrast, we found that auditory only appeared to be not sufficient to influence an attentional task in the left hemispace, while only the multisensory stimulation influenced the attention of the users and significantly improve their performance. For future research, a more heterogeneous population (i.e., with a balanced gender and different cultural background) will allow to better understand eventual aspects related to gender or cultural differences.

### Opportunities and Implications for Neuropsychology

Although we considered only participants without attentional or visuospatial deficits in this experiment, the motivation for this research was also driven by the interest of the authors in promoting VR as a tool for neuropsychological assessments and rehabilitation. While there is a range of classical methods available for clinical applications, VR and multisensory technology provide compelling and novel opportunities not yet fully exploited. With VR implementation and the introduction of olfactory stimulation for the Bells Test, we only make a small but first step toward that effort contributing to impact beyond HCI.

Several rehabilitation methods have been developed for USN, each of them with a specific theoretical framework (Pierce and Buxbaum, 2002; Azouvi et al., 2017). In the last decades, rehabilitation and assessment methods have been upgraded to deploy new technologies, and VR has already been used in neuropsychology for rehabilitation and assessment purposes such as visuospatial impairments, attention deficits, and USN (Rose et al., 2005; Pedroli et al., 2015). Moreover, VR and more specifically head-mounted displays (HMDs) had been proved to be efficient and reliable for neuropsychological purposes (Foerster et al., 2016) and to be accessible also through wheelchairs (Hansen et al., 2019). Focusing on USN treatments, VR-based methods proved in some cases to be even more sensitive in the assessment, especially for mild patients who may not be detected by standard paper-and-pencil test (Kim et al., 2011), while other studies showed better outcomes after VR-based rehabilitation sessions compared with classical methods (Buxbaum et al., 2008; Dawson et al., 2008). Moreover, multisensory stimulation is increasingly recognized and adopted as an effective USN rehabilitation method (Zigiotto et al., 2020), and VR technology constitutes the perfect tool to control and integrate different sensory modalities. Nevertheless, VR implementations of USN rehabilitation methods are focused mainly on visual and auditory stimuli (Tsirlin et al., 2009), and more rarely they include haptic interactions (Baheux et al., 2006; Teruel et al., 2015). To date and to the best knowledge of the authors, there has not been any investigation using olfactory stimuli with neuropsychological visual cancellation task, and this study represents an initial step toward the creation of VR multisensory environments that include olfactory stimulations. The results showed how multisensorial stimuli can successfully drag the visuospatial attention toward the right side of the exploration space in participants without attentive or visuospatial deficits. Future implementation of this methodology with a clinical population could provide more insights regarding the possibility of integrating smell in multisensory stimulation to drag visuospatial attention toward the neglected left side. The results obtained represent the first step into exploring the use of smell and sound in a traditional visuospatial test (i.e., the Bells Test) in a VR environment. This innovation could lead to the introduction of new rehabilitation tools that can be used for clinical purposes and open up new opportunities for working with patients.

### Spatial Attention in Human-Computer Interaction

Recent years have seen a growing interest and efforts to move HCI toward multisensory interaction (Obrist et al., 2016). The opportunities for multisensory experience design (Velasco and Obrist, 2020) and to enhance the sense of presence through smell in virtual environments are increasingly recognized [e.g., Season Traveler (Ranasinghe et al., 2018)], and yet we are only starting to scratch the surface of its potential, especially when it comes to the spatial features for smell stimulation (Maggioni et al., 2020).

This study contributes insights into the integration of olfactory and multisensory stimulation in VR, opening up a design space for a range of application scenarios. Let the following be imagined: everyday life is pervaded by notifications (e.g., emails, telephone calls, social media updates). Although notifications aim to provide information related to background events, they can also cause frustration and decrease the performance of a user by interrupting an ongoing activity (be it work, play, or learning), as each notification asks for attention. A prior study has shown that olfactory notifications can improve the performance of users and are perceived as less disruptive (Maggioni et al., 2018). While this prior study (Maggioni et al., 2018) demonstrates the benefits of olfactory stimulation, it also offers initial evidence that smell influences visuospatial attention on a computer screen in a work environment. These findings, combined with the results from this study on spatial attention, can inspire future studies on how an olfactory notification system can help guide the attention of workers toward a specific task in increasingly distributed and ubiquitous information spaces, [e.g., multiple screens, multi-platform, and cross-media contents (Brudy et al., 2019)]. To achieve the best possible performance, multimodal stimuli can be carefully crafted based on needs, preferences, and individual characteristics and backgrounds of users (e.g., handedness, cultural background). This is becoming even more relevant in light of advances in augmented reality applications, blending the real world with virtual worlds (e.g., Hartmann et al., 2019).

Moreover, outside the attentive domain, multisensory stimulations can have the additional advantage of enhancing and compensating for sensory capabilities of people, creating more inclusive interactive environments. For example, a prior study has shown that augmenting the environment with auditory and tactile stimuli can improve spatial learning and orientation in students who are blind (Albouys-Perrois et al., 2018), and has improved spatial skills in children (Brule et al., 2016). The addition of smell stimuli to virtual environments could help to increase the sense of presence of users and, above all, be used to prepare and train people in dangerous situations, such as during fire evacuations (Nilsson et al., 2019) or to recover from traumatizing experiences or posttraumatic stress disorder (PTSD), such as war scenarios (Aiken and Berry, 2015).

## Conclusions

We explored the use of smell and its combination with other modalities (sound) in a visuospatial exploration task in VR. We aimed to understand the effect of single sensory and multisensory stimulation on catching the attention of users comparing smell, sound, and smell and sound stimuli. The results showed that olfactory stimuli combined with auditory stimuli were significantly more effective in capturing the visuospatial attention of the participants toward the right side of the VR exploration space, while stimulation from the left side resulted in a higher performance toward the left hemispace, with no significant differences between the three experimental conditions. Furthermore, the VR implementation provides insights on the exploration path used to explore visuospatial stimuli. Future studies could investigate personal and cultural differences between participants, comparing performances of non-clinical vs. clinical groups (e.g., people with a diagnosis of spatial neglect due to a brain lesion). Finally, this study extends recent efforts around multisensory HCI with the potential to create not just more immersive but also more inclusive interactive experiences. Above all, we believe in the importance of integrating the sense of smell in future HCI scenarios to drive the spatial attention of participants.

## Data Availability Statement

The raw data supporting the conclusions of this article will be made available by the authors, without undue reservation.

## Ethics Statement

The studies involving human participants were reviewed and approved by the Sciences & Technology Cross-Schools Research Ethics Committee at the University of Sussex. The project reference number is ER/EM443/3. The patients/participants provided their written informed consent to participate in this study.

## Author Contributions

ND identified the potential research area in the intersection of visual attention and multisensory stimuli (i.e., use of scent and sound stimuli) through discussions with EM and MO. ND carried out a detailed literature review and defined the experimental design based on the co-authors feedback. ND developed the VR environment with support from DP and designed the experimental protocol and integration of the sensory stimuli. ND was responsible for data collection and analysis with close input and feedback from the co-authors, mainly EM, MO, and AG. All authors contributed to the article and approved the submitted version.

## Conflict of Interest

DP is employed by the company Ultraleap Ltd., but the research was carried out as part of his PhD at the University of Sussex. The remaining authors declare that the research was conducted in the absence of any commercial or financial relationships that could be construed as a potential conflict of interest. The reviewer MC declared a shared affiliation with one of the authors ND to the handling editor at time of review.
