# Corrigendum: May I Smell Your Attention: Exploration of Smell and Sound for Visuospatial Attention in Virtual Reality

**DOI:** 10.3389/fpsyg.2021.749419

**Published:** 2021-08-20

**Authors:** Nicolò Dozio, Emanuela Maggioni, Dario Pittera, Alberto Gallace, Marianna Obrist

**Affiliations:** ^1^Politecnico di Milano, Department of Mechanical Engineering, Milan, Italy; ^2^Sussex Computer-Human Interaction Lab, Department of Informatics, University of Sussex, Brighton, United Kingdom; ^3^Department of Computer Science, University College London, London, United Kingdom; ^4^Ultraleap Ltd., Bristol, United Kingdom; ^5^Mind and Behavior Technological Center - MibTec, University of Milano-Bicocca, Milan, Italy

**Keywords:** virtual reality, smell, sound, multisensory, visuospatial attention

In the published article, there was an error regarding the affiliation**(s)** for Dario Pittera. Instead of having affiliation(s) ^**^2,3,4^**^, they should only have ^**^2,4^**^.

The authors apologize for this error and state that this does not change the scientific conclusions of the article in any way. The original article has been updated.

## Publisher's Note

All claims expressed in this article are solely those of the authors and do not necessarily represent those of their affiliated organizations, or those of the publisher, the editors and the reviewers. Any product that may be evaluated in this article, or claim that may be made by its manufacturer, is not guaranteed or endorsed by the publisher.

